# CD200 Receptor Controls Sex-Specific TLR7 Responses to Viral Infection

**DOI:** 10.1371/journal.ppat.1002710

**Published:** 2012-05-17

**Authors:** Guruswamy Karnam, Tomasz P. Rygiel, Matthijs Raaben, Guy C. M. Grinwis, Frank E. Coenjaerts, Maaike E. Ressing, Peter J. M. Rottier, Cornelis A. M. de Haan, Linde Meyaard

**Affiliations:** 1 Department of Immunology, University Medical Center Utrecht, Utrecht, The Netherlands; 2 Virology Division, Department of Infectious Diseases and Immunology, Faculty of Veterinary Medicine, Utrecht University, Utrecht, The Netherlands; 3 Department of Pathobiology, Faculty of Veterinary Medicine, Utrecht University, Utrecht, The Netherlands; 4 Department of Medical Microbiology, University Medical Center Utrecht, Utrecht, The Netherlands; McMaster University, Canada

## Abstract

Immunological checkpoints, such as the inhibitory CD200 receptor (CD200R), play a dual role in balancing the immune system during microbial infection. On the one hand these inhibitory signals prevent excessive immune mediated pathology but on the other hand they may impair clearance of the pathogen. We studied the influence of the inhibitory CD200-CD200R axis on clearance and pathology in two different virus infection models. We find that lack of CD200R signaling strongly enhances type I interferon (IFN) production and viral clearance and improves the outcome of mouse hepatitis corona virus (MHV) infection, particularly in female mice. MHV clearance is known to be dependent on Toll like receptor 7 (TLR7)-mediated type I IFN production and sex differences in TLR7 responses previously have been reported for humans. We therefore hypothesize that CD200R ligation suppresses TLR7 responses and that release of this inhibition enlarges sex differences in TLR7 signaling. This hypothesis is supported by our findings that *in vivo* administration of synthetic TLR7 ligand leads to enhanced type I IFN production, particularly in female *Cd200^−/−^* mice and that CD200R ligation inhibits TLR7 signaling *in vitro*. In influenza A virus infection we show that viral clearance is determined by sex but not by CD200R signaling. However, absence of CD200R in influenza A virus infection results in enhanced lung neutrophil influx and pathology in females. Thus, CD200-CD200R and sex are host factors that together determine the outcome of viral infection. Our data predict a sex bias in both beneficial and pathological immune responses to virus infection upon therapeutic targeting of CD200-CD200R.

## Author Summary

Immune responses need to be carefully orchestrated to prevent disease due to an overactive immune system. Immunological checkpoints are provided by immune inhibitory receptors, which set a threshold for activation and dampen the immune system. In the case of a viral infection, this prevents pathology induced by the immune system, but on the other hand may prevent adequate removal of the virus. In this paper, we show that removal of such an immunological checkpoint in mice leads to rapid removal of corona virus, but also to more immune-induced disease symptoms in case of influenza virus infection. We observe this predominantly in female mice. We demonstrate that this particular checkpoint inhibits anti-viral responses that are naturally stronger in females. Release of this checkpoint enlarges these sex differences. Our findings have major implications for therapeutic use of blockers of this pathway, which are currently in clinical trials for the treatment of cancer, as we predict that female patients will have a stronger response to such therapeutics.

## Introduction

To generate an appropriately controlled response during infections, the immune system is balanced by the action of activating and inhibitory receptors. Lack of inhibition leads to excessive inflammation and autoimmunity and other severe disease symptoms. One of the receptors regulating this balance is CD200 Receptor (CD200R) [1]. CD200R was originally described as a myeloid receptor [2], being expressed on macrophages, granulocytes and DCs, but later we and others recognized that it is also expressed on T cells, B cells and NK cells [3], [4]. The CD200R intracellular domain is devoid of the classical immunoreceptor tyrosine-based inhibition motif (ITIM) present in most immune inhibitory receptors but it does have three tyrosine residues that can be phosphorylated, one of which is embedded in an NPXY motif. CD200R-downstream signaling is dependent on the recruitment of Dok2 and RasGAP [5].

The signal that triggers CD200R and results in delivery of an inhibitory intracellular signal to the cell is given by its ligand CD200, which has a short intracellular tail devoid of any known signaling motifs. CD200 is expressed on thymocytes, activated T cells, B cells, dendritic cells (DCs), vascular endothelial cells, hair follicular cells, in the central nervous system and in the retina (reviewed in [6]). Both in mice and humans, CD200 exclusively binds to the inhibitory CD200R. In contrast to humans, the mouse CD200R family contains several activating receptors, but these do not bind CD200 [7].

*Cd200^−/−^* mice were first described to be more susceptible to autoimmune disorders [8]. Later its role in microbial infections was recognized. Infection of *Cd200^−/−^* mice with the gram negative *N. meningitides* causes increased lethality, proinflammatory cytokine production and lymphocyte activation [9]. We and others showed that in mouse influenza A virus infection CD200-deficiency aggravates immune pathology [10], [11]. These studies were exclusively performed in female mice. They indicate that CD200-CD200R signaling controls the strength of the initial anti-microbial response and the return to homeostasis. We here studied the influence of CD200-CD200R blockade on clearance and pathology in two different virus infection models, coronavirus and influenza virus, in both male and female mice.

Mouse hepatitis coronavirus (MHV) is an accepted model for the most illustrious coronavirus (CoV): severe acute respiratory syndrome (SARS)-CoV. Host control of MHV infection is completely dependent on an immediate type I IFN response, initiated upon TLR7 triggering by viral RNA. Mice lacking this pathway show massive MHV replication and fatal infection within a few days [12], [13]. As a model where a strong anti-viral response causes immune mediated pathology we studied influenza A virus infection in which immune pathology is known to be important for clinical outcome.

We here report that lack of CD200R signaling has a more profound effect on the beneficial but also on the pathological immune responses to viruses in female mice as compared to male mice, which can be attributed to the capacity of CD200R to inhibit TLR7 responses.

## Results/Discussion

### CD200-deficiency and sex determine the outcome of MHV infection

**Figure 1:**
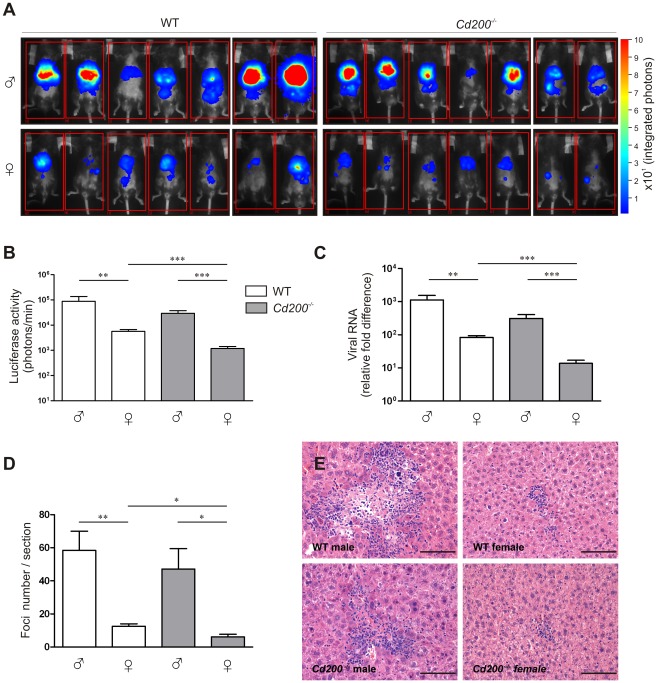
CD200-deficiency and sex determine the outcome of MHV infection. **A** Male and female WT (open bars) and *Cd200^−/−^* (gray bars) mice (n = 7 per group) were intraperitoneally inoculated with CoV (MHV-EFLM). At day 4 after infection mice were injected intraperitoneally with luciferin and were subjected to BLI. Integrated light intensity is shown. Results are representative of three independent experiments. Uninfected mice did not give a BLI signal. **B** Quantification of the data in (A). **C** Four days after infection mice were sacrificed, RNA was isolated from livers and fold-difference in viral RNA was measured by qRT-PCR. **D** Livers from mice 4 days post-infection were fixed and stained with hematoxylin and eosin. Cellular infiltration foci were quantified in the whole liver sections, (n = 7). In (B–D) mean ± SEM is shown. Statistical significance was calculated with a Mann-Whitney test. ns = not significant. * = p<0.05. ** = p<0.01. *** = p<0.005. **E** Representative example of single foci from livers of mice 4 days after infection, (n = 7). Similar results were obtained in 3 independent experiments.

To determine the role of CD200-CD200R signaling in CoV infection, we intraperitoneally inoculated male and female wild type (WT) and *Cd200^−/−^* mice with a recombinant MHV encoding luciferase (MHV-EFLM). We monitored viral spread using bioluminescence imaging (BLI) at day 2 and 4 after infection [14], [15]. Interestingly, at both time points we observed a decreased viral spread in female WT mice when compared to males. Moreover, lack of CD200 resulted in a significantly lower level of viral replication in females (**Figure 1A,B** and **Figure S1A,B,C**). The viral RNA load in the livers at day 4 was assessed by quantitative PCR and confirmed the imaging results: WT female mice had significantly lower viral RNA levels than WT male mice (**Figure 1C**). Again, CD200-deficiency greatly decreased virus load in female mice. This was confirmed in histological liver sections stained with a monoclonal antibody against MHV (data not shown). The number of focal lesions in the liver, typical for MHV was also lower in female mice and again CD200-deficiency had a significant effect on these lesions only in female mice (**Figure 1D,E**).

**Figure 2:**
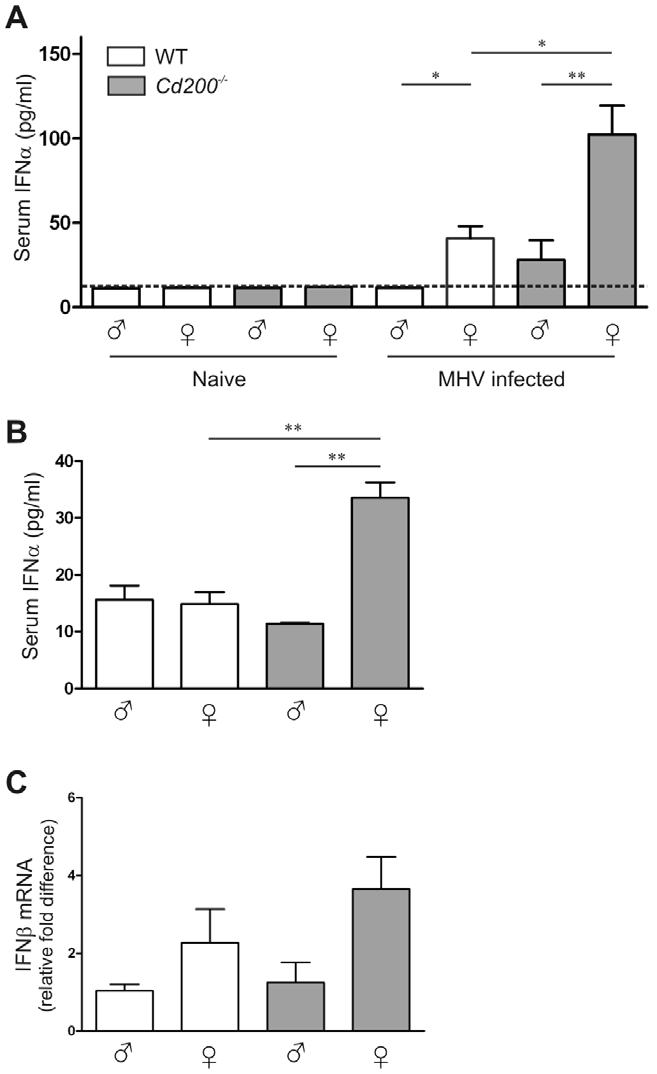
Enhanced type I interferon responses in female *Cd200^−/−^* mice. **A** IFN-α concentrations in the sera of male and female WT (open bars) and *Cd200^−/−^* (gray bars) mice were measured by ELISA. Naïve mice (n = 4) and mice at 4 days post-infection with coronavirus (MHV-EFLM) (n = 7) were used. **B** Male and female WT (open bars) and *Cd200^−/−^* (gray bars) mice were injected intraperitoneally with the TLR7 ligand imiquimod (50 µg/mouse). One-hour later sera were collected, and the concentrations of IFNα were measured by ELISA (n = 6). Dotted lines in (A,B) indicate the detection limit of the ELISA. **C** Male and female WT (open bars) and *Cd200^−/−^* (gray bars) mice were injected intraperitoneally with the TLR7 ligand imiquimod (50 µg/mouse). Twenty-four hours later livers were collected and IFNβ expression was determined by qRT-PCR (n = 6). Mean ± SEM is shown. Statistical significance was calculated with Mann-Whitney test. ns = not significant. * = p<0.05. ** = p<0.01. *** = p<0.005.

Clearance of MHV critically depends on TLR7-mediated type I IFN production by hematopoietic cells [12], [13]. In WT mice, MHV infection resulted in detectable IFN-α production only in female mice (**Figure 2A**). In *Cd200^−/−^* animals, all females and 2 out of 8 males produced detectable amounts of IFN-α and *Cd200^−/−^* female mice produced the highest amounts of IFN-α (**Figure 2A**). IFN-α concentrations in serum inversely correlated with viral load at day 4 (p = 0.04) (data not shown). Thus, the combination of female sex and CD200 deficiency results in increased type I IFN production and decreased viral load and pathology upon MHV infection.

### Enhanced TLR7 responses in female *Cd200^−/−^* mice

Sex differences in TLR7 responses have previously been reported for humans [16], [17]. Our observed sex difference in IFN-α production and viral clearance upon MHV infection in mice is likely due to a similar sex bias in TLR7 responses. We hypothesized that CD200R signaling suppresses TLR7 responses in WT mice. Release of this inhibition would further reveal the intrinsically higher TLR7 responses in females and result in more rapid viral clearance. To test this hypothesis, we first administered a TLR7 ligand *in vivo*. As described previously [18], intraperitoneal injection of a synthetic TLR7 ligand (imiquimod) leads to rapid release of type I IFN into the circulation. One-hour after injection we only detected significant amounts of IFN-α in the sera of female *Cd200^−/−^* mice, confirming the notion that CD200R inhibits the intrinsic potential for a higher TLR7 response in females (**Figure 2B**). We sacrificed the mice at 24 hrs after ligand injection, at which time point no serum IFN-α was detectable (data not shown) but type I IFN mRNA could be detected in the livers of these mice. Again, females expressed more IFN-β mRNA than males, with CD200-deficient female mice displaying even more elevated IFN-β mRNA levels (**Figure 2C**). Thus, release of CD200-mediated inhibition leads to increased production of type I IFN in response to TLR7 ligands, particularly in female mice.

### CD200R inhibits TLR7 signaling

**Figure 3:**
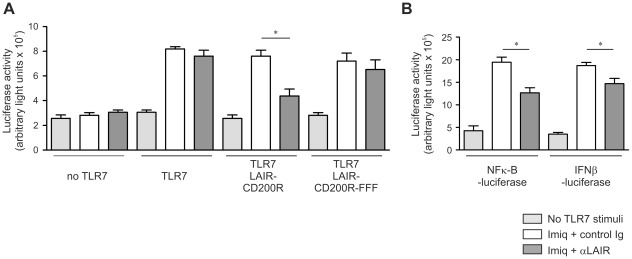
Stimulation of CD200R directly inhibits TLR mediated NFκB activity. **A** HEK 293 T cells were transiently transfected with TLR7, NF-κB luciferase reporter and LAIR-1-CD200R chimera or signaling-defective LAIR-1-CD200R-FFF constructs. Twenty-four hours later cells were stimulated with control or anti-LAIR-1 antibody. Forty-eight hours after transfection cells were stimulated with the TLR7 ligand imiquimod (3.0 µg/ml). Seventy-two hours after transfection cells were harvested, luciferase activity, and total protein content were determined. Protein normalized, luciferase activity from 3 independent experiments is shown. **B** HEK 293 T cells with stable expression of TLR7 were transiently transfected with a NF-κB luciferase reporter construct or an IFNβ luciferase reporter construct and a LAIR-1-CD200R chimera. TLR7 stimulation and CD200R crosslinking was performed as in (A). Protein normalized with luciferase activity from 3 independent experiments is shown. Mean ± SEM is shown. Statistical significance was calculated with Mann-Whitney test. ns = not significant. * = p<0.05.

We next tested whether CD200R-mediated signaling directly inhibits signals transduced via TLR7. We generated a chimeric construct containing a LAIR-1 receptor in which the intracellular tail was replaced by that of CD200R. This allows for efficient cross-linking using anti-LAIR-1 antibodies to induce signaling via the CD200R cytoplasmic tail. We transfected HEK 293 cells with plasmids encoding a LAIR-1-CD200R chimeric receptor, human TLR7 and a luciferase reporter under control of a NF-κB driven promoter. Cross-linking of the chimeric receptor by anti-LAIR-1 antibody, but not by isotype-matched control antibody, resulted in robust inhibition of imiquimod-induced NF-κB activity (**Figure 3A**). CD200R contains three intracellular tyrosine residues. A chimeric LAIR-1-CD200R protein in which all three tyrosines are mutated to phenylalanine (FFF) did not suppress TLR7 responses upon cross-linking, indicating that the observed inhibitory effect is indeed dependent on CD200R-signaling (**Figure 3A**). In a cell line with stable ectopic expression of TLR7 and transient expression of the NF-κB-reporter and the LAIR-1-CD200R constructs we also observed that TLR7 signaling was inhibited through CD200R-mediated signaling. A similar effect was observed when luciferase expression was driven by an IFN-β promoter (**Figure 3B**). Thus, the enhanced type I IFN production and viral clearance of MHV in female *Cd200^−/−^* mice can be explained by the release of CD200R-mediated inhibition of the intrinsically higher TLR7 responses in females.

### Sex-bias in viral clearance and pathology in influenza virus infected mice

**Figure 4:**
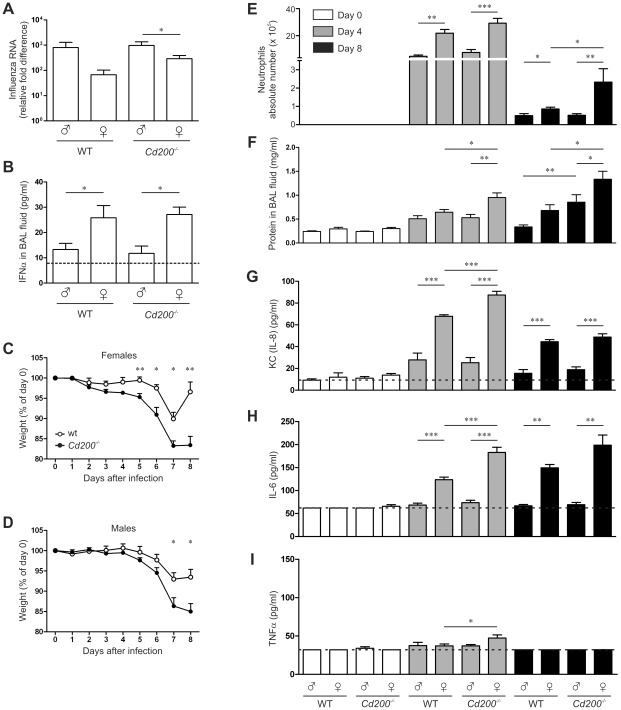
CD200-deficiency and sex determine the outcome of influenza A virus infection. Naïve or influenza A virus-infected mice were sampled at indicated time points. **A** Relative amounts of viral RNA in the lungs were determined by qRT-PCR. **B** IFNα concentration in the BAL fluid was determined by ELISA. The dotted line indicates the detection limit. **C, D** Body weight of infected female (**C**) and male (**D**) *Cd200^−/−^* mice (filled symbols) and WT mice (open symbols) as percentage of the weight at day of infection. **E** Quantification of neutrophil numbers in BAL fluid by differential cell count. **F** Quantification of the total protein content in BAL fluid. Concentrations of KC (IL-8) (**G**), IL-6 (**H**) and TNFα (**I**) in the BAL fluid were measured by ELISA. In all panels mean ± SEM is shown, statistical significance was calculated with Mann-Whitney test. * = p<0.05, ** = p<0.01.

A strong anti-viral response can also cause immune mediated pathology that can be detrimental to the host. We therefore moved to a virus infection model in which immune pathology is known to be important for clinical outcome. Upon intranasal infection with influenza A virus we again observed a sex bias in the viral load, measured in the lungs at day 8 post infection (**Figure 4A**). Female mice had lower viral loads compared to male mice, which was accompanied by enhanced IFN-α concentrations in the bronchoalveolar lavage (BAL) fluid (**Figure 4B**). However, as opposed to MHV infection, CD200-deficiency did not enhance type I IFN production in influenza virus infection (**Figure 4B**).

Confirming previous reports, we found a significantly enhanced body weight loss in female *Cd200^−/−^* mice compared to WT females (**Figure 4C**) [11], [10]. Although male *Cd200^−/−^* mice lost more weight than WT males, the weight loss started later and the difference was not as prominent (**Figure 4D**). This may indicate that lack of CD200 results in a more severe pathology in females. We observed an increased level of cellular infiltration in lung tissue of females (**Figure S2A**). Therefore we determined lung cellular influx by differential cell count in BAL fluid (**Figure S2B–D**). The total number of cells in BAL fluid was higher in all female groups but differences were not significant (**Figure S2B**). The number of lymphocytes was increased in females of both genotypes (**Figure S2D**). At day 4 after infection, the number of lymphocytes was increased in females of both WT and *Cd200−/−* groups. At day 8, the number of neutrophils in the BAL fluid was decreased in all groups, still the WT females displayed elevated numbers over males. Importantly, lack of CD200 resulted in significantly higher neutrophil counts in females but not in males (**Figure 4E**). As an additional parameter of lung damage we measured the total protein content in the BAL fluid. Lack of CD200 resulted in increased protein levels in the BAL fluid of both males and females, especially at day 8 after infection (**Figure 4F**). Overall these data indicate that female mice experience increased lung pathology upon influenza A virus infection, which is aggravated by the lack of the CD200R-regulatory pathway.

In agreement with the increased neutrophil counts we measured elevated levels of KC (IL-8) in the BAL fluid (**Figure 4G**). IL-6 concentrations were increased in all females at 4 and 8 days after infection, which was further enhanced by the lack of CD200 only at day 4 (**Figure 4H**). TNF-α was hardly detectable, but significantly increased levels were measured in *Cd200^−/−^* female mice at day 4 after infection (**Figure 4I**).

Thus, from two different viral infection models we can conclude that sex has a profound effect on type I IFN production and viral clearance. This study is the first to report a significantly enhanced viral clearance in female mice due to a sex bias in TLR7 responses. Sex differences in TLR7 induced type I IFN production have previously been reported for humans [16], [17] and our data show that in mice this has a strong impact on the course of a viral infection. The mechanism for this is not understood. On the one hand, incomplete inactivation of the *Tlr7* gene, on the X chromosome, resulting in higher TLR7 expression in females has been proposed [19], [20]. In our experiments, TLR7 mRNA expression was equal in male and female mice (**Figure S3**). This is consistent with a prior report in which no evidence for escape from X-inactivation of the *Tlr7* gene in humans was found [16]. There are conflicting reports concerning the influence of sex hormones on TLR7 responses [16], [17]. Alternatively, sex-dependent epigenetic mechanisms may contribute [16].

We demonstrate direct inhibition of TLR7 signaling through CD200R. Previously, CD200R-mediated inhibition of LPS-induced cytokine production was reported [5], [21], [22]. This suggests that CD200R affects proximal events in the TLR signaling pathway. CD200R is a unique inhibitory receptor, since its intracellular tail does not contain ITIMs. CD200R does contain three intracellular tyrosine residues. Mutation of all three tyrosine completely abrogates its inhibitory function [23]. The most distal tyrosine is located in an NPXY motif, to which the adaptor molecule Dok2 is recruited [24]. Dok 2 activates RasGAP and knockdown of these proteins diminishes the inhibitory action of CD200R [5]. However, the down-stream targets for CD200R mediated inhibition are not yet identified.

Upon influenza A virus infection, CD200-deficiency strongly enhances neutrophil influx into the lungs of female mice possibly leading to pathology, but it does not affect viral clearance and type I IFN production. This implies that, for influenza virus, the sex-biased type I IFN production and viral clearance are not regulated by CD200R, while the events leading to increased neutrophil recruitment and lung pathology are. Neutrophil responses to influenza virus infection were shown to be dependent on TLR7 [25]. Since neutrophils express CD200R, the strongly increased neutrophil influx in female *Cd200^−/−^* mice is in line with our finding that CD200R inhibits sex-biased TLR7 responses. In contrast to MHV infection, clearance of influenza A virus is not dependent on plasmacytoid dendritic cells [26]. Although influenza RNA triggers TLR7 [27], the main source of type I IFN is the infected respiratory epithelium [28]. These cells do not express CD200R and hence are not influenced by CD200-deficiency, explaining the lack of effect of CD200-deficiency on type I IFN production.

There is emerging evidence that tumor cells employ immunological checkpoints for their benefit. As a result of this, inhibitory immune pathways have become therapeutic targets to strengthen anti-tumor responses and develop (adjuvant) therapeutic strategies in cancer treatment. The successful application of anti-CTLA4 (Cytotoxic T-Lymphocyte Antigen 4) in melanoma is followed up with blocking agents for other checkpoints, among which the CD200-CD200R immune inhibitory pathway. Strong evidence for a role for CD200 in tumor progression comes from studies in patients. Expression of CD200 is an independent prognostic factor for multiple myeloma and acute myeloid leukemia predicting worse overall survival of these patients [29], [30]. A clinical trial with a blocking anti-CD200 antibody aims to enhance anti-tumor responses towards CD200-expressing malignancies (ClinicalTrials.gov Identifier: NCT00648739). On the basis of our data, one of the predicted side effects would be severe immune pathology to infections.

Our finding that the combination of lack of CD200R signaling and female sex has such a profound impact on the control of virus infection as well as on immune pathology raises some important issues. We are the first to demonstrate a strong sex bias in type I IFN production and viral clearance in mice utilizing two different models of virus infection. This is of importance for scientists studying these widely used models and may result in a completely different interpretation of data obtained, depending on the sex of the mice used. Moreover, sex biased clinical responses to virus infections have been reported in humans [31]. For influenza A virus (H5N1) a significantly enhanced case-fatality rate was found in women [32]. In agreement with these findings we now show increased lung damage, enhanced neutrophil influx and elevated IL-8, IL-6 levels in BAL fluid of female mice upon influenza A virus infection. A few reports discuss the possibility of a sex bias in severity of SARS-CoV infection [33], [34]. Also for HIV-1 infection, sex-related differences have been well-established [35], [36]. Our results underscore the importance of the issue of sex bias in scientific research, clinical trails, and vaccine studies, previously raised by others [19], [37]. Particularly, CD200 blocking antibodies are currently entering clinical trials for cancer treatment. Our data point to a possible pathological outcome of e.g. influenza virus infection in women as a result of CD200 blocking therapies.

## Materials and Methods

### Mice and viruses

Wild-type C57BL/6J mice and *Cd200^−/−^* mice, which were made and maintained on a full C57BL/6J background [8], were bred at the Specified Pathogen Free (SPF) unit at the Utrecht University Central Animal Laboratory and used between 8 and 10 weeks of age. Mice were injected intraperitoneally with 10^6^ TCID_50_ of MHV strain A59 expressing the firefly luciferase (FL) reporter gene (MHV-EFLM) [14] in 200 µl PBS. Intranasal infection with 3.0×10^4^ TCID_50_ of influenza strain A/HK/2/68 was performed as described [11]. Mice were monitored once every 24 hours for symptoms of illness.

In additional experiments we injected the mice intraperitoneally with the TLR7 agonist imiquimod (Invivogen; 50 µg in 200 µl PBS). The Utrecht University Ethical Committee for Animal Experimentation approved the animal study protocols, in accordance with the advice of the Central Committee on Animal Experimentation (20 januari 1997) and the Dutch Law on Animal Experimentation (art. 18a).

### Bioluminescence imaging

After MHV-EFLM injection (day 0), mice were imaged at day 2 and day 4 as described previously [14] with minor modifications. Briefly, mice were anaesthetized with isoflurane and subsequently injected with 100 µl of the FL substrate D-luciferin (Synchem Laborgemeinschaft OHG) dissolved in PBS (25 mg/kg). Mice were positioned to the ventral side in a specially designed box and placed onto the stage inside the light-tight photon imager (Biospace Laboratory). Five mice were imaged simultaneously exactly 5 min after the injection of D-luciferin. The bioluminescence signals were acquired with PhotoVision software (Biospace Laboratory) over a 10-min interval and are expressed as integrated light intensity (photons/min). A low-intensity visible light image was generated and used to create overlay (heatmap) images for each individual animal.

### Tissue homogenization and isolation of total RNA

Whole livers or left lungs were dissected from the mice. The tissues were processed in Lysing Matrix D tubes (MP Biomedical), containing 1 ml of PBS, using a FastPrep instrument (MP Biomedical). The tissues were homogenized at 3300× g for 40 sec and immediately placed on ice. Subsequently, the homogenates were centrifuged at 18600× g for 10 minutes at 4°C and supernatants were harvested and stored at −80°C. Total RNA was isolated from the homogenates using the TRIzol reagent (Invitrogen) according to manufacturer's instructions.

### Quantitative RT-PCR

Gene expression levels of IFN-α, IFN-β1, and TLR7 were measured by quantitative PCR using LightCycler 480 RNA Master Hydrolysis Probes in combination with a LightCycler 480 system (both from Roche Applied Science), according to the manufacturer's instructions. The housekeeping gene GAPDH was used as a reference in all experiments, and expression of this gene was found relatively constant among samples. The amounts of MHV RNA were determined by quantitative RT-PCR using primers and probe directed against the N gene of MHV-A59 [38]. For the influenza RNA quantification the primers mapping to the influenza A nucleoprotein (N) gene were used. Amplification and detection were performed with an ABI Prism 7700 system. Samples were controlled for the presence of possible inhibitors of the amplification reaction by internal control (murine encephalomyocarditis virus DNA).

### Bronchoalveolar lavage analysis

Bronchoalveolar lavage (BAL) fluid was obtained by flushing the lungs two times with 1 ml PBS using a canula inserted into the trachea, yielding around 1.7 ml BAL fluid. Pelleted cells from BAL fluid were counted and cytospins were prepared and stained with May-Grunwald/Giemsa and neutrophils were scored on the basis of morphology (Dade Behring, Switzerland). BAL fluids were kept on ice or stored at −80°C until further processing. BAL fluid was centrifuged, and 20 µl of aliquot was used to determine the protein concentration with a BCA kit (Pierce) according to the manufacturer's instructions.

### Cytokine analysis

To measure the interferon concentration in the sera, blood was sampled from naïve mice or four days after MHV infection or one hour after imiquimod treatment. Sera were separated by spinning the blood at 2300× g for 15 minutes at 4°C. For measurement of cytokines in BAL, samples were prepared by spinning 5 minutes at 530× g. IFN-α was measured with a mouse interferon alpha ELISA kit (PBL Interferon Source). For the IL-6, IL-8 and TNF-alpha measured by Mouse IL-6 Mni ELISA Development Kit, Murine KC (IL-8) ELISA Development Kit and Murine TNF-alpha Mini ELISA Development Kit (PeproTech) respectively. Experiments were done according to manufacturer's instructions.

### Liver histopathology

Livers of MHV-infected mice were sampled, fixed in 4% neutral buffered formalin, and embedded in paraffin. Seven µm liver sections were stained with hematoxylin and eosin. Total liver sections were examined by light microscopy and foci of hepatocellular necrosis and inflammation were scored in a semi-quantitative manner.

### Reporter assays

HEK 293 T cells were transiently co-transfected with: human TLR7 (kindly provided by Rogier Sanders, AMC, Amsterdam, the Netherlands), and NF-κB-reporter or IFNα-reporter constructs, kindly provided by Dr Paul Moynagh (National University of Ireland). A chimeric construct containing the extra cellular region of human LAIR-1 (amino acids 1–160) fused with the transmembrane and intracellular rat CD200R (rCD200R) (amino acids 236–327) was cloned into pcDNA3.1/zeo (Invitrogen, Breda, the Netherlands). A tyrosine (Y) to phenylalanine (F) mutant of tyrosines 287, 290, and 298 in the intracellular rCD200R tail were generated with PCR-based mutagenesis. The mutant was cloned into the same vector and all sequences were confirmed by automated DNA sequencing. The LAIR-CD200R plasmid was co-transfected with the TLR7 and reporter constructs. Twenty-four hours after transfection cells were trypsinized and seeded in 48-well plates coated with 3 ug/ml anti-LAIR-1 monoclonal antibody (clone 8A8). Forty-eight hrs after transfection cells were stimulated with imiquimod 3 µg/ml (Invivogen) in PBS. On the next day, cells were lysed with Passive Lysis Buffer (Promega), luciferase activity was measured on a luminometer (Berthold technologies Centro LB 960), and data were analyzed with Microwin software. Total protein content was determined with a Pierce BCA Protein Assay (Thermo Scientific). All luciferase values were normalized to protein concentration. Alternatively, we used HEK 293 cells stably expressing human TLR7 (Invivogen).

### Statistical analysis

Significance was calculated with Mann-Whitney test using GraphPad Prism software.

### Online supplemental material

Figure S1 shows BLI measurement in naïve mice and viral spread of MHV at day 2 after infection. Figure S2 depicts the quantification of lung pathology and differential cell count in the BAL fluid in influenza A virus infected mice. Figure S3 is a quantitative analysis of TLR7 mRNA in male and female mice.

## Supporting Information

Figure S1 **CD200-deficiency and sex determine the outcome of MHV infection.** (**A**) Uninfected WT and *Cd200^−/−^* mice were injected intraperitoneally with luciferin and were subjected to bioluminescence imaging (BLI). Presented pictures are integrated images of normal light picture and luciferase signal. (**B**) At day 2 after infection with CoV (MHV-EFLM) mice were injected intraperitoneally with luciferin and were subjected to BLI. Integrated light intensity is shown. Results are representative of three independent experiments. (**C**) Quantification of the data in (B). Mean ± SEM is shown.
(DOC)

Figure S2 **Sex determines lung pathology and cellular infiltration after influenza infection.** (**A**) At day 8 after influenza infection lungs were sampled and H&E stained. The nuclear lung surface area was used as a benchmark of the inflammatory response, its color range selections were measured thrice. The relative nuclear surface area (nuclear surface area/lung surface area * 100) of the lung sections was used as a measure of the tissue response to exposure to the influenza virus (**B**) Total cell count in BAL fluid. Quantification of monocyte (**C**) and lymphocyte (**D**) numbers in BAL fluid by differential cell count. In all panels mean ± SEM is shown, statistical significance was calculated with Mann-Whitney test. * = p<0.05, ** = p<0.01.
(DOC)

Figure S3 **No sex difference in expression of TLR7 mRNA.** Four days after MHV injection mice were sacrificed, RNA was isolated from livers and TLR7 mRNA expression was quantified by qPCR in male and female WT and *Cd200^−/−^* mice. Mean ± SEM is shown.
(DOC)
