# Supplementary material for: CD200 Receptor Controls Sex-Specific TLR7 Responses to Viral Infection
Source: PLoS Pathog. 2012 May 17;8(5):e1002710. doi: 10.1371/journal.ppat.1002710 (PMC3355091; doi:10.1371/journal.ppat.1002710)
Supplement: Figure S1 — CD200-deficiency and sex determine the outcome of MHV infection. (A) Uninfected WT and Cd200−/− mice were injected intraperitoneally with luciferin and were subjected to bioluminescence imaging (BLI). Presented pictures are integrated images of normal light picture and luciferase signal. (B) At day 2 after infection with CoV (MHV-EFLM) mice were injected intraperitoneally with luciferin and were subjected to BLI. Integrated light intensity is shown. Results are representative of three independent experiments. (C) Quantification of the data in (B). Mean ± SEM is shown. (DOC) [file ppat.1002710.s001.doc]

**
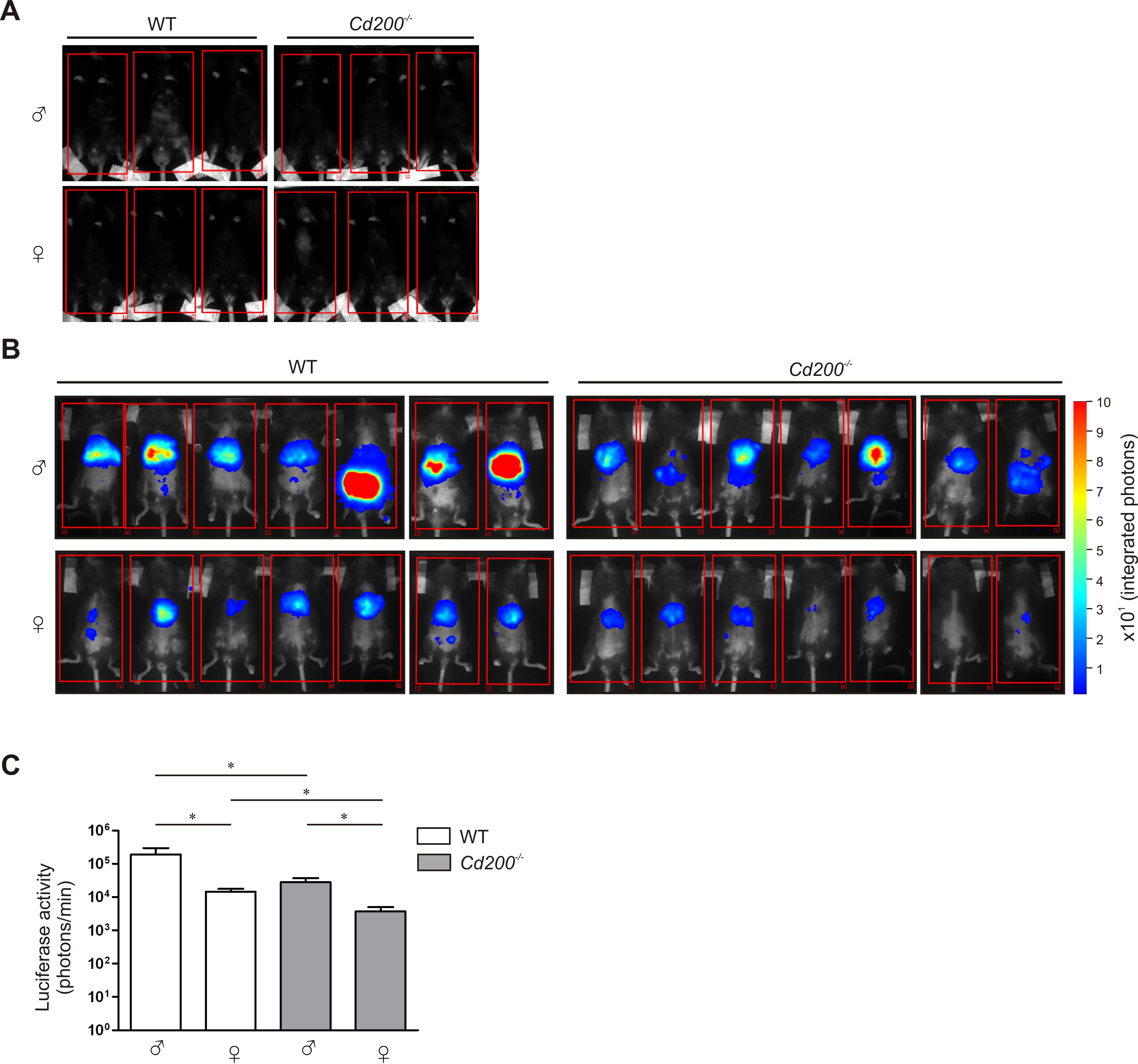
**

**Figure S1. CD200-deficiency and sex determine the outcome of MHV infection. (A)** Uninfected WT and *Cd200^-/-^* mice were injected intraperitoneally with luciferin and were subjected to bioluminescence imaging (BLI). Presented pictures are integrated images of normal light picture and luciferase signal. **(B)** At day 2 after infection with CoV (MHV-EFLM) mice were injected intraperitoneally with luciferin and were subjected to BLI. Integrated light intensity is shown. Results are representative of three independent experiments. **(C)** Quantification of the data in (B). Mean ± SEM is shown.
