# Supplementary material for: CD200 Receptor Controls Sex-Specific TLR7 Responses to Viral Infection
Source: PLoS Pathog. 2012 May 17;8(5):e1002710. doi: 10.1371/journal.ppat.1002710 (PMC3355091; doi:10.1371/journal.ppat.1002710)
Supplement: Figure S2 — Sex determines lung pathology and cellular infiltration after influenza infection. (A) At day 8 after influenza infection lungs were sampled and H&E stained. The nuclear lung surface area was used as a benchmark of the inflammatory response, its color range selections were measured thrice. The relative nuclear surface area (nuclear surface area/lung surface area * 100) of the lung sections was used as a measure of the tissue response to exposure to the influenza virus (B) Total cell count in BAL fluid. Quantification of monocyte (C) and lymphocyte (D) numbers in BAL fluid by differential cell count. In all panels mean ± SEM is shown, statistical significance was calculated with Mann-Whitney test. * = p<0.05, ** = p<0.01. (DOC) [file ppat.1002710.s002.doc]

**
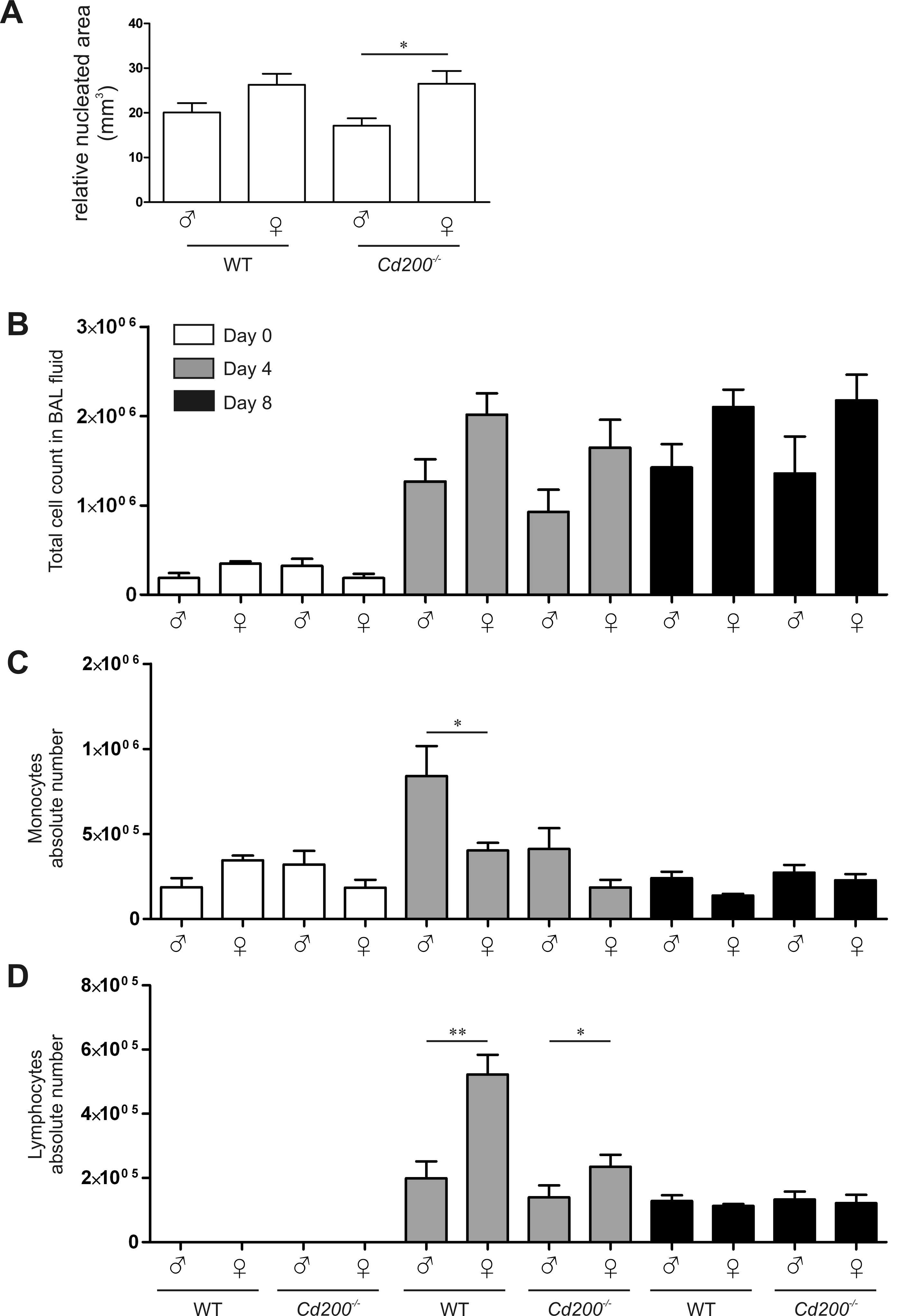
**

**Figure S2. Sex determines lung pathology and cellular infiltration after influenza infection. (A)** At day 8 after influenza infection lungs were sampled and H&E stained. The nuclear lung surface area was used as a benchmark of the inflammatory response, its color range selections were measured thrice. The relative nuclear surface area (nuclear surface area / lung surface area * 100) of the lung sections was used as a measure of the tissue response to exposure to the influenza virus **(B)** Total cell count in BAL fluid. Quantification of monocyte **(C)** and lymphocyte **(D)** numbers in BAL fluid by differential cell count. In all panels mean ± SEM is shown, statistical significance was calculated with Mann-Whitney test. * = p<0.05, ** = p< 0.01.
