# Supplementary material for: CD200 Receptor Controls Sex-Specific TLR7 Responses to Viral Infection
Source: PLoS Pathog. 2012 May 17;8(5):e1002710. doi: 10.1371/journal.ppat.1002710 (PMC3355091; doi:10.1371/journal.ppat.1002710)
Supplement: Figure S3 — No sex difference in expression of TLR7 mRNA. Four days after MHV injection mice were sacrificed, RNA was isolated from livers and TLR7 mRNA expression was quantified by qPCR in male and female WT and Cd200−/− mice. Mean ± SEM is shown. (DOC) [file ppat.1002710.s003.doc]

**
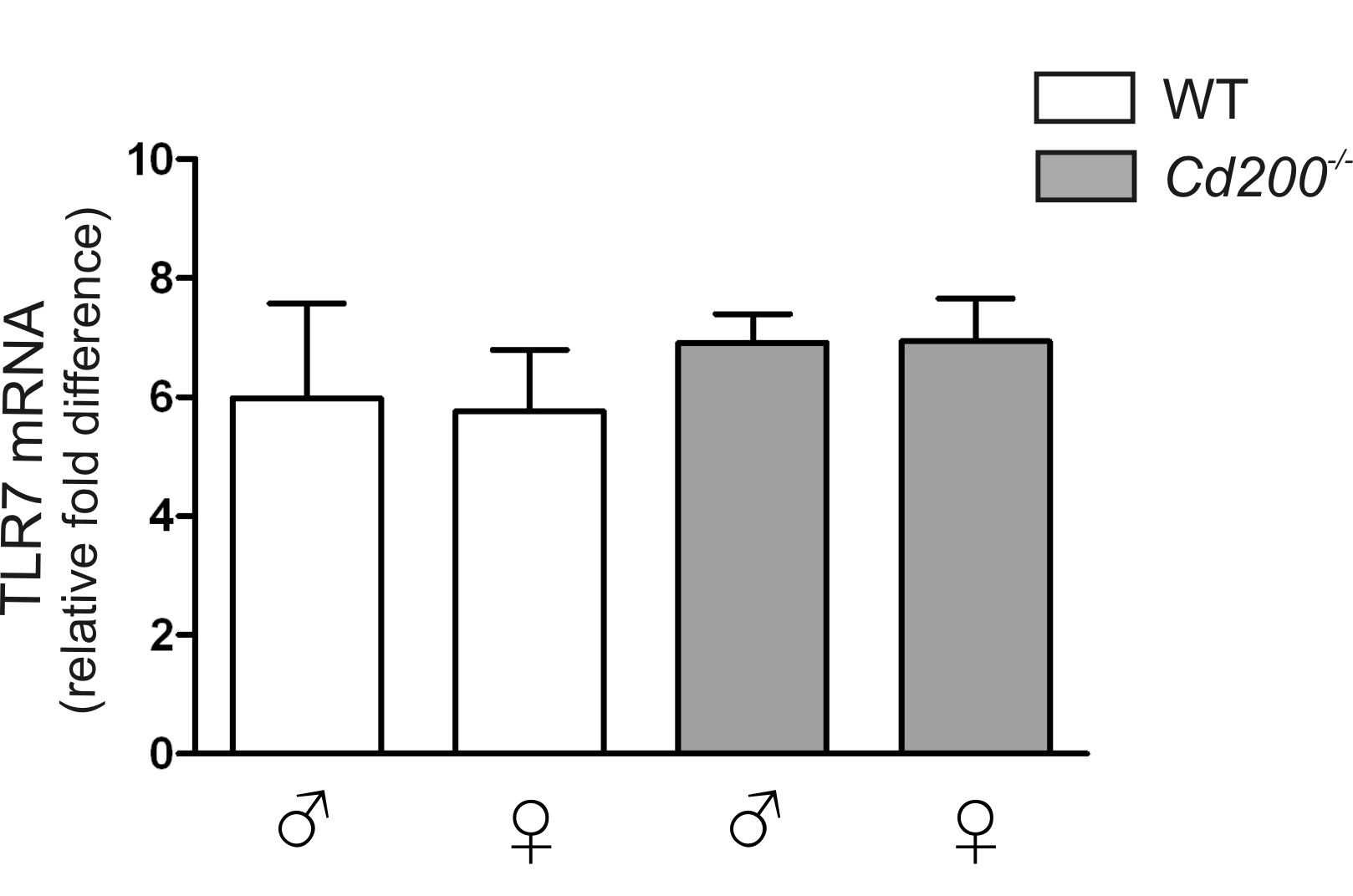
**

**Figure S3. No sex difference in expression of TLR7 mRNA**

Four days after MHV injection mice were sacrificed, RNA was isolated from livers and TLR7 mRNA expression was quantified by qPCR in male and female WT and *Cd200^-/-^* mice. Mean ± SEM is shown.
